# Health problems associated with single, multiple, and the frequency of months of objectively measured long working hours: a cohort study by the National Institute of Occupational Safety and Health, Japan

**DOI:** 10.1007/s00420-021-01768-x

**Published:** 2021-10-20

**Authors:** Yuko Ochiai, Masaya Takahashi, Tomoaki Matsuo, Takeshi Sasaki, Yuki Sato, Kenji Fukasawa, Tsuyoshi Araki, Yasumasa Otsuka

**Affiliations:** 1 National Institute of Occupational Safety and Health, 6-21-1Tama-ku, Kawasaki, Nagao, 214-8585 Japan; 2 Advantage Risk Management Co., Ltd., Tokyo, Japan; 3 Faculty of Human Sciences, University of Tsukuba, Tokyo, Japan

**Keywords:** Overtime, Exposure assessment, Health checkup, Low-density lipoprotein cholesterol

## Abstract

**Purpose:**

We aimed to examine the prospective associations of monthly working hours measured in a month, the 6-month averaged hours, and the frequency of long working hours (≥ 205 h/month) during the past 6 months with health indicators.

**Methods:**

This study included 6,806 Japanese company workers (response rate = 86.6%). Data on the workers’ monthly attendance during the second half of fiscal year 2016 and annual health checkups in fiscal years 2016 and 2017 were collected. We evaluated the association of the above three types of monthly working hours with subsequent health checkup data in fiscal year 2017. We adjusted for the corresponding data in fiscal year 2016.

**Results:**

Multivariate logistic regression analyses revealed significant associations between monthly working hours and workers’ systolic and diastolic blood pressure as well as aspartate aminotransferase, alanine aminotransferase, low-density lipoprotein cholesterol (LDL), and triglyceride levels. However, the associations were not consistent between months. The average monthly working hours were significantly associated with higher LDL levels for the 220–240 h/mo group (OR: 1.49, 95%CI: 1.07–2.08) and lower triglyceride levels for the < 140 h/mo group (OR: 0.15, 95%CI: 0.03–0.77), compared to the 140–180 h/mo group. The frequency of long working hours was significantly associated with higher LDL levels.

**Conclusions:**

Working hours over several months produced various associations with health indicators compared to those measured in a single month. Our present data suggest that the effects of average or frequency of long working hours during the past 6 months are likely to appear in LDL levels.

## Introduction

The issue on long working hours has been seriously discussed since the late 1970s in Japan. This is because working long hours has led to the deaths of some Japanese workers (Takahashi 2019). The term *karoshi*, which means death due to overwork, is now commonly used worldwide. In Japan, cerebrovascular and cardiovascular diseases are recognized as work-related and workers who developed these diseases are compensated when (1) overtime work exceeds 100 h a month before the disease onset or (2) 80 h per month from 2 to 6 months prior to the disease onset (Japan Ministry of Health, Labor and Welfare 2018a). In spite of the government’s long standing efforts to prevent overwork-related diseases, health problems still occur due to overwork. In 2020, 113 people with cerebrovascular disease and 81 people with ischemic heart disease were recognized as having overwork-related physical diseases. Currently, East Asian countries/regions, such as South Korea and Taiwan, have similar standards for recognizing work-related injuries (Chang and Lin 2019).

In the field of occupational epidemiology research, long working hours are considered to cause physical and psychological health problems in workers. These include the metabolic syndrome (Kobayashi et al. 2012; Pimenta et al. 2015), high blood pressure (Nakamura et al. 2012; Trudel et al. 2020), cerebrovascular and cardiovascular diseases (Virtanen and Kivimäki 2018; Hayashi et al. 2019; Lin et al. 2018), sleep disturbances (Virtanen et al. 2009), depression (Virtanen et al. 2012; Kim et al. 2016), and work-related injuries (Yamauchi et al. 2019). Numerous studies have evaluated the relationship between working hours and health problems. However, these studies presented inconsistent conclusions. In 2020, two meta-analyses were published (Li et al. 2020; Descatha et al.^2020^). Li et al. (2020) concluded that the risk of stroke incidence and mortality resulting from long working hours over 55 h/week had a moderate quality of evidence, whereas incidence and mortality resulting from long working hours (from 41 h/week to 54 h/week) had a low quality of evidence. Descatha et al. (2020) concluded that the risk of ischemic heart disease due to long working hours had a low quality of evidence (mortality) and moderate quality of evidence (incidence). The conclusions of both studies were based on studies that measured the weekly and/or daily working hours using self-reported questionnaires or face-to-face interviews (Li et al. 2020; Descatha et al. 2020). However, in 2021, another systematic review from the World Health Organization and International Labor Organization concluded that exposure to long working hours (≥ 55 h/week) caused a large attributable burden of ischemic heart disease and stroke (Pega et al. 2021).

Although the association between long working hours and workers’ health has been studied for an extended period, to the best of our knowledge, the measurement of working hours through exposure assessment has not been explored in detail. Majority of previous studies relied on workers’ self-report of the number of hours worked within 1 month (i.e., the month prior to the beginning of those studies) (Takahashi 2019; Kobayashi et al. 2012; Nakamura et al. 2012; Trudel et al. 2020). However, some workers may not be able to accurately report their working hours because of lapses in memory or they may intentionally provide false responses; this emphasizes the utility of objectively measured working hours (e.g., attendance records), as highlighted in our previous study (Ochiai et al. 2020). In addition, the number of working hours measured within a single time period cannot provide information regarding the working hours during the preceding period. Commonly, working hours can vary on a monthly basis (Carraher and Parnell 2002). Moreover, we have not been able to assess the number of intervals of working long hours in a given period. In other words, it seems impossible to evaluate the “frequency” of exposure to long working hours based on workers’ health status. However, one previous study used the workers’ attendance records for 3 months to assess the same. The exposure factor was determined as whether the worker had worked for more than 205 h. The study examined the effects of long hours of overtime on the workers’ health (Nishikitani et al. 2005). Nevertheless, this study did not consider the frequency of exposure to long working hours. That is, it did not consider how *often* the workers had been exposed to long working hours. Thus, we propose that assessing the working hours from an objective and long-term perspective is necessary to understand the influence of working hours on health.

As mentioned above, most previous studies have measured the working hours as a workplace exposure factor in a given month. The three objectives of this study were follows: (1) to examine the difference in the association between monthly attendance data (objectively measured) and the outcome (workers’ health), (2) to examine the difference in the association between total (average) working hours and workers’ health as opposed to the association between each month’s working hours and workers’ health, and (3) to examine the association between the frequency of exposure to long working hours and workers’ health. In other words, we aimed to examine the association between the frequency of exposure to long working hours of ≥ 45 h/mo over a 6-month period [Japan's newly established overtime limit (Japan Ministry of health, Labor and Welfare 2018b)] and workers’ health indicators.

## Methods

### Participants

Workers of a Japanese tertiary sector company were invited to participate in this cohort study by the National Institute of Occupational Safety and Health, Japan (Ochiai et al. 2020). These workers included managerial, sales, clerical, and product workers, among others. Of the 7,857 workers, 6,806 agreed to participate (response rate = 86.6%). We gathered the workers’ information from the monthly attendance management system of the personnel department in the second half of 2016. The workers’ electronic ID cards were swiped in the attendance management system, and information regarding the time at which the workers entered and left the office were collected from the system. In addition, we conducted an online questionnaire survey in 2017 and collected the workers’ sociodemographic factors for this study. All participants provided their written informed consent online after receiving information regarding the purpose and process of the study.

### Measures

#### Monthly working hours and their averages within the second half of the fiscal year 2016

The monthly working hours were measured according to the corresponding values indicated in the attendance records for monthly working hours during the second half of the fiscal year 2016, specifically the time period from October 2016 to March 2017 as most Japanese corporations end their fiscal year at the end of March. The average monthly working hours were calculated for this same period. The workers were divided into six groups according to their monthly working hours: > 140 h/mo group (equivalent to > 35 h/wk), 140–180 h/mo group (35–45 h/wk), 180–205 h/mo group (45–51.25 h/wk), 205–220 h/mo group (51.25–55 h/wk), 220–240 h/mo group, 55 h–60 h/wk), and ≥ 240 h/mo group (60 h/wk). Workers with more than 3 months of working hour data were included in the analysis.

#### Frequency of exposure to long working hours within 6 months

We defined long working hours as the monthly working hours exceeding the legal limit of 205 h per month. Its frequency was measured from October 2016 to March 2017 (i.e., zero, once, twice,…, six times). The inclusion criteria used for calculating the monthly working hours were applied to determine the frequency of exposure to long working hours.

#### Health checkups

Annual health checkups for the workers were conducted in fiscal years 2016 and 2017. Data on the following indicators were obtained during the checkups sex, age, body mass index (BMI), blood pressure (systolic and diastolic), liver function (aspartate aminotransferase [AST], alanine aminotransferase [ALT], and gamma-glutamyl transpeptidase [GGT] levels), high-density lipoprotein (HDL) and low-density lipoprotein (LDL) cholesterol levels, and triglyceride levels. The following results were considered as abnormal findings: BMI ≥ 25 kg/m^2^, systolic blood pressure ≥ 140 mmHg, diastolic blood pressure ≥ 90 mmHg, AST level ≥ 30 IU/L, ALT level ≥ 30 IU/L, GGT level ≥ 50 IU/L, HDL cholesterol level ≤ 40 mg/dL, LDL cholesterol level ≥ 120 mg/dL, and triglyceride level ≥ 150 mg/dL. The health status in 2016 was used as an adjustment variable. The health indicators used as the outcome were data from the health checkup conducted in 2017 (from April 2017 to March 2018).

#### Covariates

The online questionnaire measured the participants’ employment type, job category, and work schedule. To obtain a variable reflecting the previous health status, we determined if the health indicators from the checkups conducted in fiscal year 2016 yielded abnormal findings according to the health criterion values (true = 1, false = 0). Moreover, we calculated the time period from the end of the previous fiscal year (March 2017) to the health checkup conducted in the next fiscal year (any month starting from April 2017) to create a metric controlling for the effect of the interval between the two measurements. The unit was defined as the number of months included in the exposure period. If the health checkup was conducted in May 2017, the elapsed time was counted as 2 months.

### Statistical analyses

The differences among the workers in the various working-hour categories (in terms of the values assigned for the demographic variables and health indicators) were examined using the χ^2^ test. The average and standard deviation values for each indicator were determined. Binominal logistic regression analyses were conducted to determine the associations between the monthly working hours and the health indicators. We also analyzed the association between the average monthly working hours within 6 months and the health data. Two models were set. The first model was adjusted for sex, age, time period until the health checkup in fiscal year 2017, employment type, job category, and work schedule (Model 1). The same model (Model 1) was adjusted for the presence of abnormal findings in order to set the second model (Model 2). Similarly, binominal logistic regression analyses were used to determine the odds ratios (ORs) and their corresponding 95% confidence intervals (CIs) for the associations between the frequency of exposure to long working hours (i.e., zero to six times) and workers’ health. The workers who had not worked long hours were considered as the reference group (i.e., zero). The workers for whom the values of the variables were missing were excluded from the analyses. All analyses were performed using the IBM® SPSS® Statistics (version 26).

## Results

### Demographic characteristics of the study participants

In Model 1, the study included 4,177 men and 1,969 women (total number = 6,146). The mean ages were 37.7 years (SD = 9.6 years) for men and 36.3 years (SD = 11.3 years) for women. In Model 2, data of 3,817 men (mean age, 37.9 [SD = 8.8]) and 1,325 women (mean age, 34.0 [SD = 10.0]) were analyzed. Table 1 shows the characteristics of participants by average work hour category included in Model 2. Table 2 shows the characteristics of participants by frequency of exposure to long working hours included in Model 2. Within 6 months, 2,357 workers (45.8%) were classified as the 180–205 h/mo group. A total of 937 (18.2%) workers had not worked for more than 205 h within a 6-month period at any given time. For all six groups categorized based on the average working hours, the mean time period from the end of the previous fiscal year to the date of health checkup (months) was 4.8–5.2 months (SD: 1.4–1.8 mo). For all seven groups categorized based on the frequency of exposure to long working hours, the mean time period from the end of the previous fiscal year to the date of health checkup (months) was 4.8– 5.0 months (SD: 1.3–1.4 mo).

**Table 1:** Characteristics of the workers according to the average monthly working hours in the second half of 2016 (Model 2)

		Average monthly working hours in the second half of 2016												
		< 140 h		140–180 h		180–205 h		205–220 h		220–240 h		≧ 240 h		
		*n*	(%)	*n*	(%)	*n*	(%)	*n*	(%)	*n*	(%)	*n*	(%)	*p*
Sex														< 0.01
	Men	8	(20)	224	(52.5)	1675	(71.1)	1122	(80.3)	567	(81.8)	221	(97.4)	
	Women	32	(80)	203	(47.5)	682	(28.9)	276	(19.7)	126	(18.2)	6	(2.6)	
Age, years														< 0.01
	≤ 29	4	(10)	88	(20.6)	697	(29.6)	409	(29.3)	121	(17.5)	-	-	
	30–39	23	(57.5)	161	(37.7)	758	(32.2)	595	(42.6)	362	(52.2)	144	(63.4)	
	40–49	8	(20)	107	(25.1)	606	(25.7)	297	(21.2)	175	(25.3)	76	(33.5)	
	≥ 50	5	(12.5)	71	(16.6)	296	(12.6)	97	(6.9)	35	(5.1)	7	(3.1)	
Job category														< 0.01
	Manager	4	(10)	68	(15.9)	462	(19.6)	284	(20.3)	219	(31.6)	185	(81.5)	
	Non-managerial	36	(90)	359	(84.1)	1895	(80.4)	1114	(79.7)	474	(68.4)	42	(18.5)	
Employment type														< 0.01
	Regular employee	38	(95)	375	(87.8)	2176	(92.3)	1365	(97.6)	689	(99.4)	227	(100)	
	Non-regular workers^2^	2	(5)	52	(12.2)	181	(7.7)	33	(2.4)	4	(0.6)	-	-	
Work schedule														< 0.01
	Fixed time system	36	(90)	371	(86.9)	1873	(79.5)	1148	(82.1)	582	(84)	181	(79.7)	
	Variable working hours system	3	(7.5)	33	(7.7)	257	(10.9)	170	(12.2)	77	(11.1)	37	(16.3)	
	Flextime system	0	(0)	12	(2.8)	87	(3.7)	45	(3.2)	14	(2)	5	(2.2)	
	Discretionary labor system	1	(2.5)	0	(0)	11	(0.5)	6	(0.4)	8	(1.2)	3	(1.3)	
	Shift system, others	-	-	11	(2.6)	129	(5.5)	29	(2.1)	12	(1.7)	1	(0.4)	
Total		40	(100)	427	(100)	2357	(100)	1398	(100)	693	(100)	227	(100)	
Time period from the end of the previous fiscal year to the date of health checkup (months), mean (SD)^1^		5.2	(1.8)	4.8	(1.4)	4.8	(1.3)	4.8	(1.4)	4.9	(1.4)	5.1	(1.4)	
Frequency of exposure to work hours of over 205 h per month within 6 months, mean (SD)		0.2	(0.5)	0.2	(0.5)	1.4	(1.0)	4.0	(1.0)	5.5	(0.6)	6.0	(0.1)	
BMI														< 0.01
	< 25 kg/m2	34	(85.0)	329	(77.0)	1,729	(73.4)	974	(69.7)	495	(71.4)	158	(69.6)	
	≧ 25 kg/m2	6	(15.0)	98	(23.0)	628	(26.6)	424	(30.3)	198	(28.6)	69	(30.4)	
Systolic blood pressure														< 0.05
	< 140 mmHg	37	(92.5)	396	(93.0)	2,175	(92.3)	1,306	(93.4)	665	(96.0)	213	(93.8)	
	≧ 140 mmHg	3	(7.5)	30	(7.0)	182	(7.7)	92	(6.6)	28	(4.0)	14	(6.2)	
Diastolic blood pressure														0.32
	< 90 mmHg	37	(92.5)	392	(92)	2,186	(92.7)	1,320	(94.4)	650	(93.8)	209	(92.1)	
	≧ 90 mmHg	3	(7.5)	34	(8)	171	(7.3)	78	(5.6)	43	(6.2)	18	(7.9)	
AST														0.06
	< 30 IU/L	36	(90.0)	383	(90.1)	2,073	(88.2)	1,199	(85.9)	597	(86.4)	189	(83.6)	
	≧ 30 IU/L	4	(10.0)	42	(9.9)	278	(11.8)	196	(14.1)	94	(13.6)	37	(16.4)	
ALT														< 0.01
	< 30 IU/L	34	(85.0)	341	(80.2)	1,811	(77.1)	985	(70.6)	479	(69.3)	152	(67.3)	
	≧ 30 IU/L	6	(15.0)	84	(19.8)	539	(22.9)	410	(29.4)	212	(30.7)	74	(32.7)	
GGT														< 0.01
	< 50 IU/L	36	(90.0)	358	(84.4)	1,887	(80.2)	1,093	(78.4)	515	(74.5)	165	(73.0)	
	≧ 50 IU/L	4	(10.0)	66	(15.6)	465	(19.8)	301	(21.6)	176	(25.5)	61	(27.0)	
HDL cholesterol														0.06
	≧ 40 mg/dL	38	(97.4)	394	(92.9)	2,160	(92.0)	1,276	(91.6)	629	(91.8)	193	(86.5)	
	< 40 mg/dL	1	(2.6)	30	(7.1)	189	(8.0)	117	(8.4)	56	(8.2)	30	(13.5)	
LDL cholesterol														< 0.01
	< 120 mg/dL	29	(72.5)	284	(66.8)	1,465	(62.4)	775	(55.7)	361	(52.2)	106	(47.1)	
	≧ 120 mg/dL	11	(27.5)	141	(33.2)	883	(37.6)	617	(44.3)	330	(47.8)	119	(52.9)	
Triglyceride														< 0.01
	< 150 mg/dL	38	(95.0)	347	(81.6)	1,863	(79.2)	1,103	(79.1)	516	(74.9)	149	(66.2)	
	≧ 150 mg/dL	2	(5.0)	78	(18.4)	490	(20.8)	291	(20.9)	173	(25.1)	76	(33.8)	

^1^For example, the time period from the end of the exposure period (March 2017) to the date of health checkup in next May is counted as 2 months

^2^Non-regular workers include contract workers, fixed-term employees, temporary staff, and part-time workers

*AST* aspartate aminotransferase; *ALT* alanine aminotransferase; *BMI* body mass index; *GGT* gamma-glutamyl transpeptidase; *HDL* high density lipoprotein; *LDL* low density lipoprotein

**Table 2:** Characteristics of the workers according to the frequency of monthly long working hours during the second half of 2016 (Model 2)

		Frequency of long working hours^1^														
		Zero		Once		Twice		Three times		Four times		Five times		Six times		
		*n*	(%)^3^	*n*	(%)	*n*	(%)	*n*	(%)	*n*	(%)	*n*	(%)	*n*	(%)	*p*
Sex^2^																< 0.01
	Men	506	(54.0)	646	(70.2)	578	(80.2)	489	(80.8)	500	(79.1)	521	(77.9)	577	(87.7)	
	Women	431	(46.0)	274	(29.8)	143	(19.8)	116	(19.2)	132	(20.9)	148	(22.1)	81	(12.3)	
Age, years																< 0.01
	≤ 29	226	(24.1)	279	(30.3)	243	(33.7)	182	(30.1)	168	(26.6)	156	(23.3)	65	(9.9)	
	30–39	312	(33.3)	298	(32.4)	243	(33.7)	240	(39.7)	269	(42.6)	327	(48.9)	354	(53.8)	
	40–49	229	(24.4)	249	(27.1)	163	(22.6)	130	(21.5)	157	(24.8)	132	(19.7)	209	(31.8)	
	≥ 50	170	(18.1)	94	(10.2)	72	(10.0)	53	(8.8)	38	(6.0)	54	(8.1)	30	(4.6)	
Job category																< 0.01
	Manager	148	(15.8)	190	(20.7)	143	(19.8)	114	(18.8)	139	(22.0)	146	(21.8)	342	(52.0)	
	Non-managerial	789	(84.2)	730	(79.3)	578	(80.2)	491	(81.2)	493	(78.0)	523	(78.2)	316	(48.0)	
Employment type																< 0.01
	Regular employee	813	(86.8)	850	(92.4)	694	(96.3)	585	(96.7)	614	(97.2)	658	(98.4)	656	(99.7)	
	Non-regular workers^4^	124	(13.2)	70	(7.6)	27	(3.7)	20	(3.3)	18	(2.8)	11	(1.6)	2	(0.3)	
Work schedule																< 0.01
	Fixed time system	726	(77.5)	760	(82.6)	589	(81.7)	510	(84.3)	526	(83.2)	554	(82.8)	526	(79.9)	
	Variable working hours system	114	(12.2)	81	(8.8)	75	(10.4)	57	(9.4)	70	(11.1)	82	(12.3)	98	(14.9)	
	Flextime system	26	(2.8)	35	(3.8)	30	(4.2)	20	(3.3)	17	(2.7)	17	(2.5)	18	(2.7)	
	Discretionary labor system	5	(0.5)	3	(0.3)	3	(0.4)	2	(0.3)	3	(0.5)	4	(0.6)	9	(1.4)	
	Shift system, others	66	(6.6)	41	(4.4)	24	(3.3)	16	(2.6)	16	(2.5)	12	(1.8)	7	(1.1)	
Total		937	(100)	920	(100)	721	(100)	605	(100)	632	(100)	669	(100)	658	(100)	
Time period from the end of the previous fiscal year (March, 2017) to the date of health checkup (months), Mean (SD)^5^		4.8	(1.3)	4.8	(1.3)	4.8	(1.4)	4.8	(1.3)	4.9	(1.4)	4.9	(1.3)	5.0	(1.4)	
BMI (23.3 ± 4.1)^6^																< 0.01
	< 25 kg/m2	727	(77.6)	682	(74.1)	516	(71.6)	412	(68.1)	446	(70.6)	472	(70.6)	464	(70.5)	
	≧ 25 kg/m2	210	(22.4)	238	(25.9)	205	(28.4)	193	(31.9)	186	(29.4)	197	(29.4)	194	(29.5)	
Systolic blood pressure (116.2 ± 15.6)																0.07
	< 140 mmHg	874	(93.4)	847	(92.1)	669	(92.8)	553	(91.4)	590	(93.4)	631	(94.3)	628	(95.4)	
	≧ 140 mmHg	62	(6.6)	73	(7.9)	52	(7.2)	52	(8.6)	42	(6.6)	38	(5.7)	30	(4.6)	
Diastolic blood pressure (71.5 ± 11.8)																0.50
	< 90 mmHg	875	(93.5)	844	(91.7)	680	(94.3)	562	(92.9)	591	(93.5)	628	(93.9)	614	(93.3)	
	≧ 90 mmHg	61	(6.5)	76	(8.3)	41	(5.7)	43	(7.1)	41	(6.5)	41	(6.1)	44	(6.7)	
AST (22.7 ± 12.8)																< 0.05
	< 30 IU/L	840	(90.2)	805	(87.6)	625	(86.7)	534	(88.6)	535	(84.9)	572	(85.8)	566	(86.1)	
	≧ 30 IU/L	91	(9.8)	114	(12.4)	96	(13.3)	69	(11.4)	95	(15.1)	95	(14.2)	91	(13.9)	
ALT (27.1 ± 25.0)																< 0.01
	< 30 IU/L	758	(81.4)	706	(76.9)	531	(73.6)	433	(71.8)	445	(70.6)	468	(70.2)	461	(70.2)	
	≧ 30 IU/L	173	(18.6)	212	(23.1)	190	(26.4)	170	(28.2)	185	(29.4)	199	(29.8)	196	(29.8)	
GGT (40.9 ± 55.5)																< 0.01
	< 50 IU/L	768	(82.6)	734	(79.9)	583	(80.9)	480	(79.5)	488	(77.6)	508	(76.2)	493	(75)	
	≧ 50 IU/L	162	(17.4)	185	(20.1)	138	(19.1)	124	(20.5)	141	(22.4)	159	(23.8)	164	(25)	
HDL cholesterol (57.8 ± 14.8)																0.13
	≧ 40 mg/dL	876	(94.2)	830	(90.6)	659	(91.5)	555	(91.7)	574	(91.4)	604	(91.1)	592	(90.9)	
	< 40 mg/dL	54	(5.8)	86	(9.4)	61	(8.5)	50	(8.3)	54	(8.6)	59	(8.9)	59	(9.1)	
LDL cholesterol (115.0 ± 31.3)																< 0.01
	< 120 mg/dL	616	(66.1)	603	(65.8)	422	(58.6)	362	(59.9)	332	(52.8)	353	(53.2)	332	(50.6)	
	≧ 120 mg/dL	316	(33.9)	313	(34.2)	298	(41.4)	242	(40.1)	297	(47.2)	311	(46.8)	324	(49.4)	
Triglyceride (115.9 ± 96.6)																< 0.05
	< 150 mg/dL	758	(81.3)	729	(79.3)	561	(77.8)	482	(79.7)	496	(78.9)	500	(75.2)	490	(74.8)	
	≧ 150 mg/dL	174	(18.7)	190	(20.7)	160	(22.2)	123	(20.3)	133	(21.1)	165	(24.8)	165	(25.2)	

^1^Frequency of monthly working hours of 205 h or more within the 6-month period (from October 2016 to March 2017)

^2^Men (*n* = 3,817, mean = 37.9 ± 8.8 years old), women (*n* = 1,325, mean = 34.0 ± 10.0 years old)

^3^Figures do not always add up to 100% due to the rounding of data

^4^Non-regular workers include contract workers, fixed-term employees, temporary staff, and part-time workers

^5^For example, the time period from the end of the exposure period (March 2017) to the date of health checkup in next May is counted as 2 months

^6^Mean ± SD

*AST* aspartate aminotransferase; *ALT* alanine aminotransferase; *BMI* body mass index; *GGT* gamma-glutamyl transpeptidase; *HDL* high density lipoprotein; *LDL* low density lipoprotein

### Association of health checkup data in 2017 with the monthly working hours in each month of the second half of 2016 and with the 6-month average of the monthly workin﻿g hours

Table 3 shows the association of the health checkup data with the monthly working hours within the 6-month period. It also shows the association of the health checkup data with the average monthly working hours during the same period. In this table, the results based on Model 2 are presented only to save space. Significant associations were found between the monthly working hours in some months and abnormal systolic blood pressure, diastolic blood pressure, AST levels, ALT levels, LDL cholesterol levels, and triglyceride levels. However, these associations were not consistent during the 6-month period. The average monthly working hours within the 6-month period were positively associated with abnormal LDL cholesterol levels in a dose–response manner in the 220–240 h/mo group (OR: 1.49, 95%CI: 1.07–2.08). With regard to triglyceride levels, the 6-month average monthly working hours were significantly associated with abnormal findings in the < 140 h/mo group (OR: 0.15, 95% CI: 0.03–0.77). The other health indicators were not significantly associated with working hours.

**Table 3:** Association of the health-checkup data from 2017 with the monthly working hours in the second half of 2016 and with the average monthly working hours in the same period (Model 2)

			Participants with missing values	Monthly working hours										
				< 140 h		140–180 h	180–205 h		205–220 h		220–240 h		≧ 240 h	
	Month	N		OR	(95% CI)	(Reference)	OR	(95% CI)	OR	(95% CI)	OR	(95% CI)	OR	(95% CI)
BMI														
	2016.10	5,098	1,048	0.28	(0.07—1.18)	1	0.99	(0.61—1.61)	0.99	(0.60—1.64)	1.03	(0.61—1.72)	0.95	(0.52—1.75)
	2016.11	5,114	1,032	0.61	(0.17—2.16)	1	0.97	(0.67—1.42)	0.98	(0.64—1.49)	0.95	(0.59—1.54)	1.11	(0.58—2.12)
	2016.12	5,130	1,016	0.72	(0.22—2.34)	1	1.20	(0.87—1.65)	1.38	(0.96—2.00)	1.39	(0.87—2.22)	1.18	(0.59—2.35)
	2017.1	5,142	1,004	1.14	(0.40—3.26)	1	1.07	(0.78—1.47)	1.06	(0.73—1.53)	1.07	(0.67—1.69)	0.68	(0.31—1.50)
	2017.2	5,141	1,005	0.48	(0.12—1.89)	1	0.98	(0.67—1.43)	1.04	(0.69—1.57)	1.16	(0.73—1.82)	0.64	(0.34—1.21)
	2017.3	5,141	1,005	1.00	(0.15—6.72)	1	0.94	(0.45—1.98)	1.06	(0.50—2.22)	1.33	(0.64—2.78)	0.98	(0.46—2.07)
	Average	5,142	1,004	0.42	(0.08—2.25)	1	1.00	(0.63—1.60)	1.26	(0.77—2.07)	1.20	(0.70—2.05)	0.73	(0.36—1.50)
Systolic blood pressure														
	2016.10	5,097	1,049	-	-	1	**0.52**	**(0.34—0.80)**	**0.62**	**(0.40—0.98)**	**0.45**	**(0.28—0.73)**	**0.42**	**(0.23—0.77)**
	2016.11	5,113	1,033	1.56	(0.45—5.39)	1	1.02	(0.69—1.50)	0.96	(0.62—1.49)	0.84	(0.50—1.41)	0.81	(0.39—1.67)
	2016.12	5,129	1,017	1.43	(0.46—4.50)	1	0.82	(0.61—1.12)	**0.60**	**(0.40—0.89)**	0.77	(0.47—1.27)	0.74	(0.35—1.56)
	2017.1	5,141	1,005	1.65	(0.57—4.76)	1	0.98	(0.72—1.35)	0.83	(0.56—1.24)	0.77	(0.46—1.28)	0.88	(0.37—2.06)
	2017.2	5,140	1,006	0.78	(0.19—3.15)	1	0.90	(0.62—1.30)	0.88	(0.58—1.32)	**0.60**	**(0.36—0.99)**	0.60	(0.28—1.24)
	2017.3	5,140	1,006	**5.97**	**(1.40—25.40)**	1	1.67	(0.69—4.05)	2.17	(0.90—5.27)	1.51	(0.62—3.68)	1.08	(0.43—2.70)
	Average	5,141	1,005	1.21	(0.26—5.63)	1	1.04	(0.65—1.66)	0.91	(0.55—1.49)	0.63	(0.35—1.15)	0.82	(0.38—1.74)
Diastolic blood pressure														
	2016.10	5,097	1,049	1.02	(0.20—5.18)	1	0.93	(0.57—1.52)	0.74	(0.44—1.24)	0.78	(0.45—1.33)	0.70	(0.37—1.32)
	2016.11	5,113	1,033	0.35	(0.04—2.79)	1	0.96	(0.63—1.46)	0.91	(0.57—1.45)	0.90	(0.53—1.52)	0.74	(0.36—1.50)
	2016.12	5,129	1,017	0.74	(0.15—3.61)	1	0.98	(0.70—1.37)	0.84	(0.56—1.27)	1.08	(0.66—1.76)	0.47	(0.21—1.05)
	2017.1	5,141	1,005	1.29	(0.40—4.18)	1	0.87	(0.62—1.21)	0.95	(0.63—1.43)	0.89	(0.54—1.47)	0.63	(0.26—1.53)
	2017.2	5,140	1,006	0.74	(0.19—2.94)	1	0.73	(0.49—1.07)	0.82	(0.53—1.25)	**0.60**	**(0.36—0.99)**	0.53	(0.26—1.09)
	2017.3	5,140	1,006	**5.15**	**(1.24—21.43)**	1	1.33	(0.58—3.08)	1.27	(0.55—2.93)	1.40	(0.61—3.23)	1.06	(0.45—2.50)
	Average	5,141	1,005	1.00	(0.22—4.53)	1	0.76	(0.47—1.21)	0.65	(0.39—1.09)	0.75	(0.43—1.31)	0.59	(0.28—1.25)
AST														
	2016.10	5,085	1,061	0.93	(0.25—3.47)	1	**0.67**	**(0.45—0.99)**	**0.65**	**(0.43—0.98)**	0.69	(0.46—1.04)	**0.61**	**(0.38—0.99)**
	2016.11	5,101	1,045	0.44	(0.12—1.65)	1	1.00	(0.72—1.38)	0.98	(0.68—1.41)	1.04	(0.70—1.56)	1.03	(0.61—1.74)
	2016.12	5,117	1,029	1.23	(0.45—3.38)	1	0.94	(0.72—1.22)	0.92	(0.68—1.25)	1.12	(0.77—1.63)	1.04	(0.61—1.78)
	2017.1	5,128	1,018	1.33	(0.51—3.47)	1	1.00	(0.77—1.30)	1.10	(0.81—1.50)	0.87	(0.59—1.29)	1.63	(0.93—2.86)
	2017.2	5,127	1,019	1.72	(0.61—4.89)	1	0.95	(0.69—1.32)	0.93	(0.65—1.32)	1.04	(0.71—1.53)	0.92	(0.55—1.53)
	2017.3	5,127	1,019	1.51	(0.27—8.54)	1	1.60	(0.79—3.24)	1.32	(0.65—2.67)	1.62	(0.81—3.27)	1.22	(0.60—2.48)
	Average	5,128	1,018	2.97	(0.96—9.22)	1	0.93	(0.62—1.39)	0.94	(0.62—1.43)	1.01	(0.64—1.59)	0.94	(0.53—1.68)
ALT														
	2016.10	5,084	1,062	1.06	(0.35—3.17)	1	0.99	(0.70—1.40)	0.93	(0.65—1.33)	1.17	(0.81—1.69)	0.89	(0.58—1.37)
	2016.11	5,100	1,046	1.04	(0.41—2.64)	1	1.12	(0.85—1.47)	1.15	(0.84—1.56)	**1.46**	**(1.04—2.05)**	**1.87**	**(1.20—2.91)**
	2016.12	5,116	1,030	0.96	(0.38—2.42)	1	1.15	(0.92—1.43)	1.07	(0.82—1.38)	1.34	(0.97—1.85)	1.15	(0.72—1.84)
	2017.1	5,127	1,019	0.93	(0.39—2.22)	1	0.93	(0.75—1.17)	1.05	(0.81—1.37)	1.28	(0.93—1.76)	1.30	(0.77—2.18)
	2017.2	5,126	1,020	0.71	(0.25—2.04)	1	0.84	(0.63—1.10)	0.97	(0.72—1.30)	1.16	(0.84—1.61)	1.31	(0.86—2.02)
	2017.3	5,126	1,020	0.75	(0.17—3.24)	1	1.18	(0.67—2.08)	1.02	(0.58—1.81)	1.31	(0.75—2.30)	1.43	(0.81—2.52)
	Average	5,127	1,019	2.12	(0.69—6.46)	1	0.87	(0.62—1.22)	1.03	(0.72—1.46)	1.20	(0.82—1.77)	1.35	(0.83—2.21)
GGT														
	2016.10	5,084	1,062	1.35	(0.35—5.22)	1	0.80	(0.52—1.24)	0.91	(0.58—1.42)	1.05	(0.67—1.66)	0.85	(0.50—1.43)
	2016.11	5,100	1,046	0.79	(0.23—2.67)	1	1.03	(0.73—1.47)	1.23	(0.84—1.81)	1.24	(0.81—1.91)	1.17	(0.67—2.05)
	2016.12	5,116	1,030	0.92	(0.29—2.97)	1	1.01	(0.76—1.34)	1.16	(0.84—1.60)	1.35	(0.90—2.01)	1.17	(0.66—2.07)
	2017.1	5,127	1,019	0.23	(0.04—1.33)	1	1.19	(0.90—1.57)	1.17	(0.84—1.63)	1.30	(0.87—1.94)	1.10	(0.58—2.09)
	2017.2	5,126	1,020	0.53	(0.11—2.65)	1	1.07	(0.75—1.51)	0.98	(0.67—1.42)	1.38	(0.92—2.08)	1.05	(0.61—1.82)
	2017.3	5,126	1,020	0.29	(0.05—1.68)	1	1.13	(0.56—2.30)	0.98	(0.49—1.99)	1.18	(0.59—2.38)	1.22	(0.60—2.48)
	Average	5,127	1,019	1.27	(0.27—6.05)	1	1.04	(0.67—1.61)	1.28	(0.81—2.02)	1.49	(0.91—2.44)	1.19	(0.64—2.21)
HDL cholesterol														
	2016.10	5,069	1,077	2.10	(0.45—9.76)	1	0.80	(0.46—1.39)	0.80	(0.45—1.41)	0.94	(0.53—1.67)	0.66	(0.34—1.27)
	2016.11	5,085	1,061	0.61	(0.13—2.90)	1	1.01	(0.65—1.57)	1.08	(0.66—1.75)	0.86	(0.50—1.50)	1.23	(0.62—2.44)
	2016.12	5,101	1,045	0.61	(0.13—2.87)	1	0.94	(0.67—1.33)	0.79	(0.53—1.19)	0.75	(0.45—1.24)	1.10	(0.55—2.19)
	2017.1	5,113	1,033	0.69	(0.15—3.09)	1	0.93	(0.66—1.31)	0.66	(0.43—1.01)	1.07	(0.66—1.75)	0.81	(0.34—1.92)
	2017.2	5,112	1,034	0.60	(0.12—3.10)	1	0.92	(0.60—1.42)	0.68	(0.43—1.09)	0.99	(0.59—1.64)	0.66	(0.33—1.31)
	2017.3	5,112	1,034	0.11	(0.01—1.19)	1	0.79	(0.34—1.82)	0.91	(0.40—2.07)	0.79	(0.35—1.80)	0.88	(0.38—2.02)
	Average	5,113	1,033	0.26	(0.02—3.18)	1	0.85	(0.50—1.43)	0.74	(0.43—1.29)	0.73	(0.40—1.34)	1.13	(0.54—2.35)
LDL cholesterol														
	2016.10	5,077	1,069	1.61	(0.73—3.55)	1	0.89	(0.66—1.21)	1.23	(0.90—1.69)	1.29	(0.93—1.79)	1.44	(0.98—2.11)
	2016.11	5,093	1,053	1.27	(0.63—2.56)	1	1.22	(0.96—1.55)	**1.42**	**(1.09—1.85)**	**1.51**	**(1.12—2.05)**	**1.76**	**(1.15—2.68)**
	2016.12	5,109	1,037	1.75	(0.93—3.32)	1	1.15	(0.95—1.41)	**1.46**	**(1.16—1.84)**	1.24	(0.92—1.66)	1.37	(0.88—2.13)
	2017.1	5,121	1,025	0.90	(0.47—1.72)	1	0.99	(0.81—1.20)	**1.44**	**(1.13—1.82)**	**1.52**	**(1.14—2.03)**	1.32	(0.81—2.17)
	2017.2	5,120	1,026	0.97	(0.43—2.21)	1	**1.34**	**(1.05—1.70)**	**1.53**	**(1.18—1.99)**	**1.66**	**(1.24—2.22)**	**1.72**	**(1.16—2.56)**
	2017.3	5,120	1,026	2.41	(0.82—7.07)	1	1.33	(0.85—2.08)	1.20	(0.77—1.87)	1.45	(0.93—2.26)	**1.66**	**(1.06—2.61)**
	Average	5,121	1,025	1.21	(0.47—3.11)	1	1.02	(0.77—1.36)	1.29	(0.95—1.75)	**1.49**	**(1.07—2.08)**	1.53	(0.98—2.39)
Triglyceride														
	2016.10	5,082	1,064	0.49	(0.14—1.75)	1	0.83	(0.59—1.17)	0.77	(0.54—1.10)	0.89	(0.62—1.27)	1.00	(0.66—1.50)
	2016.11	5,098	1,048	0.32	(0.10—1.04)	1	0.97	(0.74—1.27)	0.89	(0.65—1.21)	1.14	(0.81—1.60)	1.53	(0.99—2.35)
	2016.12	5,114	1,032	0.51	(0.18—1.42)	1	0.94	(0.75—1.17)	0.95	(0.73—1.23)	0.98	(0.71—1.35)	1.47	(0.94—2.29)
	2017.1	5,126	1,020	**0.33**	**(0.11—0.93)**	1	0.90	(0.72—1.13)	0.90	(0.69—1.17)	1.07	(0.78—1.47)	**1.77**	**(1.09—2.89)**
	2017.2	5,125	1,021	0.64	(0.23—1.78)	1	0.79	(0.60—1.03)	0.80	(0.60—1.07)	0.92	(0.67—1.27)	0.92	(0.60—1.40)
	2017.3	5,125	1,021	0.61	(0.15—2.54)	1	1.31	(0.76—2.26)	1.14	(0.66—1.96)	1.23	(0.72—2.12)	1.40	(0.81—2.42)
	Average	5,126	1,020	**0.15**	**(0.03—0.77)**	1	0.88	(0.63—1.22)	0.84	(0.59—1.18)	1.06	(0.73—1.55)	1.19	(0.74—1.92)

Adjusted for age, sex, job category, employment type, work schedule, and time period until the health checkups in the fiscal year 2017 and the presence of abnormal findings with respect to each health-checkup variable in 2016 (yes or no)

Boldfaced values indicate statistically significant results

### Association of health checkup data in 2017 with the frequency of monthly long working hours during the second half of 2016

Table 4 shows the association between the frequency of exposure to long working hours and health checkup data. In Model 1, exposure to long working hours three times was significantly associated with abnormal BMI, six times for abnormal systolic blood pressure, and two times or more for abnormal LDL cholesterol. In Model 2, when the associations were adjusted for the abnormal findings of each health checkup variable in the previous year, significant associations were only observed in LDL cholesterol levels [OR: 1 for zero time, OR: 1.32 (95% CI: 1.01–1.73) two times, OR: 1.60 (95%CI: 1.21–2.11) four times, OR: 1.44 (95% CI: 1.09–1.90) five times, and OR: 1.45 (95% CI: 1.10–1.93) for six times]. The other health indicators were not significantly associated with working hours.

**Table 4:** Association of health checkup data in 2017 with the frequency of monthly long working hours in the second half of 2016 (Models 1 and 2)

			participants with missing values	Frequency of long working hours^1^												
				Zero	Once		Twice		Three times			Four times	Five times		Six times	
		N		(Reference)	OR	(95%CI)	OR	(95%CI)	OR	(95%CI)	OR	(95%CI)	OR	(95%CI)	OR	(95%CI)
BMI																
	Model 1^2^	5,205	941	1	1.11	(0.89—1.38)	1.2	(0.95—1.51)	**1.41**	**(1.10—1.79)**	1.23	(0.97—1.57)	1.23	(0.97—1.56)	1.07	(0.84—1.37)
	Model 2^3^	5,142	1,004	1	1.04	(0.69—1.56)	1.14	(0.74—1.75)	1.26	(0.81—1.97)	1.12	(0.72—1.75)	1.09	(0.71—1.69)	1.20	(0.76—1.88)
Systolic blood pressure																
	Model 1	5,204	942	1	1.02	(0.71—1.44)	0.84	(0.57—1.24)	1.04	(0.71—1.54)	0.83	(0.55—1.25)	0.68	(0.45—1.04)	**0.52**	**(0.33—0.83)**
	Model 2	5,141	1,005	1	1.26	(0.84—1.89)	1.09	(0.70—1.70)	1.26	(0.80—1.97)	1.11	(0.70—1.76)	0.91	(0.57—1.46)	0.71	(0.42—1.20)
Diastolic blood pressure																
	Model 1	5,204	942	1	1.20	(0.83—1.72)	0.78	(0.51—1.19)	0.99	(0.65—1.51)	0.88	(0.57—1.34)	0.81	(0.53—1.25)	0.75	(0.49—1.16)
	Model 2	5,141	1,005	1	1.20	(0.78—1.85)	0.88	(0.54—1.44)	0.96	(0.58—1.57)	1.14	(0.70—1.85)	0.83	(0.50—1.35)	0.87	(0.53—1.44)
AST																
	Model 1	5,198	948	1	1.04	(0.77—1.40)	1.01	(0.74—1.38)	0.83	(0.59—1.16)	1.15	(0.84—1.58)	1.07	(0.78—1.46)	0.90	(0.65—1.25)
	Model 2	5,128	1,018	1	0.97	(0.69—1.37)	0.94	(0.66—1.35)	0.80	(0.54—1.18)	1.04	(0.72—1.50)	0.96	(0.67—1.39)	0.94	(0.65—1.38)
ALT																
	Model 1	5,197	949	1	0.97	(0.76—1.23)	1.02	(0.79—1.30)	1.10	(0.85—1.42)	1.17	(0.91—1.51)	1.20	(0.94—1.54)	1.01	(0.78—1.30)
	Model 2	5,127	1,019	1	0.98	(0.73—1.32)	1.05	(0.77—1.42)	1.15	(0.84—1.59)	1.11	(0.81—1.53)	1.23	(0.90—1.68)	1.24	(0.90—1.71)
GGT																
	Model 1	5,197	949	1	0.96	(0.75—1.23)	0.81	(0.62—1.06)	0.88	(0.66—1.16)	0.98	(0.75—1.28)	1.06	(0.81—1.38)	0.89	(0.68—1.17)
	Model 2	5,127	1,019	1	0.78	(0.54—1.13)	0.75	(0.51—1.10)	0.88	(0.59—1.31)	1.15	(0.78—1.70)	1.01	(0.68—1.49)	1.03	(0.69—1.53)
HDL cholesterol																
	Model 1	5,188	958	1	1.33	(0.93—1.90)	1.07	(0.73—1.58)	1.02	(0.68—1.54)	1.06	(0.71—1.59)	1.12	(0.75—1.66)	0.96	(0.64—1.43)
	Model 2	5,113	1,033	1	1.46	(0.92—2.30)	0.99	(0.60—1.63)	1.20	(0.71—2.00)	0.94	(0.56—1.57)	1.12	(0.68—1.86)	0.97	(0.58—1.62)
LDL cholesterol																
	Model 1	5,194	952	1	0.96	(0.79—1.18)	**1.30**	**(1.05—1.61)**	1.21	(0.97—1.51)	**1.65**	**(1.32—2.05)**	**1.60**	**(1.29—1.99)**	**1.52**	**(1.22—1.90)**
	Model 2	5,121	1,025	1	0.86	(0.66—1.11)	**1.32**	**(1.01—1.73)**	1.13	(0.85—1.50)	**1.60**	**(1.21—2.11)**	**1.44**	**(1.09—1.90)**	**1.45**	**(1.10—1.93)**
Triglyceride																
	Model 1	5,198	948	1	0.94	(0.74—1.20)	0.95	(0.74—1.23)	0.83	(0.63—1.09)	0.86	(0.66—1.12)	1.08	(0.83—1.39)	0.87	(0.67—1.13)
	Model 2	5,126	1,020	1	0.94	(0.71—1.26)	0.99	(0.73—1.34)	0.89	(0.64—1.22)	0.90	(0.65—1.23)	1.15	(0.85—1.56)	1.02	(0.74—1.39)

^1^Defined as the frequency of exposure to > 205 h/m working hours within the 6-month period (from October 2016 to March 2017)

^2^Adjusted for age, sex, job category, employment type, work schedule, and period until the health checkups in fiscal year 2017 (Model 1)

^3^Adjusted for Model 1 plus the presence of abnormal findings on each health checkup variable in 2016 (yes or no)

Boldfaced values indicate significant results

*AST* aspartate aminotransferase; *ALT* alanine aminotransferase; *BMI* body mass index; *GGT* gamma-glutamyl transpeptidase; *HDL* high density lipoprotein; *LDL* low density lipoprotein

## Discussion

We investigated how the three types of monthly working hours were prospectively associated with workers’ health as determined based on the annual health checkup data. The working hours measured in a single month showed different associations with the health indicators from the 6-month averaged working hours. Data from the present study indicated that the increased frequency of exposure to long working hours was significantly associated with the number of abnormal LDL cholesterol measurements. This finding was consistent even after controlling for relevant factors, such as sociodemographic factors or previous health conditions.

### Single-month working hours and workers’ health

In this study, we initially examined how the prospective associations between the monthly working hours for each month within the 6-month period and workers’ health differed from the prospective associations between the average monthly working hours within the 6-month period and workers’ health. The associations between monthly working hours in each month and health indicator values were not consistent throughout the 6-month period. Some indicators showed lower odds of having outlier health indicators in the group with longer working hours. However, these findings were similar to those of previous studies (Imai et al., 2014; Virtanen et al, 2019). These counterintuitive findings may be related to a one-time measurement of working hours. These results also remind us of the health worker effect (Chowdhury et al, 2017; Li et al., 1999). This effect implies that the participants were so healthy that they could tolerate working longer hours, which is a serious health hazard. As mentioned in the Introduction section, an adequate assessment of working hours is one of the key research issues concerning working hours and health. The results of the present study (Table 3) revealed inconsistent associations between monthly working hours in a single month and health checkup data. The fact that most previous studies measured the working hours based on the workers’ self-reports in the past month, average working hours, or the usual working hours may have resulted in mixed findings on the effects of working hours on health. To improve the quality of evidence, more effort is required to determine the working hours over a longer period of time (Carraher and Parnell 2002; Nishikitani et al. 2005). In the present study, we measured the working hours objectively and during a long-term period. However, significant associations were observed between working hours and abnormal LDL cholesterol levels among the 205–220 h/mo, 220–240 h/mo, and ≥ 240 h/mo groups in some months. The 6-month average of the monthly working hours was associated with abnormal increases in LDL cholesterol levels among the aforementioned groups of workers.

### Average of 6-month working hours and worker’s health

With regard to the 6-month averaged working hours, in the present study, both LDL cholesterol levels and triglyceride levels were significantly associated with the average monthly working hours. This result is in accordance with that of a previous cross-sectional study. This study indicated that male workers with long working hours had higher LDL cholesterol levels than those without long working hours (Virtanen et al. 2019). Another cross-sectional study suggested that long working hours (≥ 60 h/wk) may be associated with a higher risk of arteriosclerosis (Chou et al. 2015). In Model 2, many of the 2016 health checkup data of the < 140 h/mo group were missing. Therefore, they were omitted from the analysis in Model 2. Only 40 out of 98 workers in the < 140 h/mo group had available 2016 triglyceride data. Because of the insufficient number of workers who worked < 140 h/mo, we were uncertain if the triglyceride levels of the < 140 h/mo group were accurate (OR: 0.15, 95% CI: 0.03–0.77). However, increase in the working hours from 180–205 h/mo to ≥ 240 h/mo was associated with increased ORs of LDL cholesterol levels [OR: 1.02 (95% CI: 0.03–0.77), OR: 1.29 (95% CI: 0.95–1.75), OR: 1.49 (95% CI: 1.07–2.08), and 1.53 (95% CI: 0.98–2.39)]. Therefore, lipid metabolism may be affected by the averaged working hours.

### Frequency of exposure to long working hours within the past 6 months and workers’ health

We then investigated the influence of the frequency of exposure to long working hours for 6 months on workers’ health. Data from the present study indicated that the number of abnormal LDL cholesterol measurements significantly increased as the frequency of exposure to long working hours increased. This was evident if the data were adjusted for relevant factors, such as sociodemographic factors or previous health conditions.

The results of the present study indicate that frequent exposure to long working hours may have unfavorable effects on workers’ LDL cholesterol levels. The mechanisms underlying these findings remain unclear. Previous studies have found that psychological stressors increase the lipid concentrations (Brindley et al. 1993; Wirts et al. 2009). These studies explained the mechanism by which a rapid increase in cortisol and catecholamine (epinephrine and norepinephrine) levels occurs in response to acute psychological stressors. This process results in increased concentrations of fatty acids and glycerol. Another prospective study found that middle-aged men whose workload had increased during the year preceding the study period had increased LDL cholesterol levels (Siegrist et al. 1998). Apart from psychosocial factors, sleep problems, such as short or poor sleep due to long working hours, may be related to the occurrence of dyslipidemia (Korostovtseva et al. 2020; Keovanini et al. 2020). However, the currently available evidence does not support the possibility of a specific association between sleep problems and high LDL cholesterol levels (Kruisbrink et al. 2017; Abdurahman et al. 2020). Increased physical inactivity and/or sedentary behavior are also believed to affect the lipid profile of an individual. However, recent meta-analyses did not show significant changes in LDL cholesterol levels following the respective intervention (Mulchandani 2019; Hadgraft et al. 2021).

### Strengths and limitations

The present study has both strengths and limitations. We collected the attendance data within a 6-month period from a company record. Thus, we were able to exclude the possible effect of lapses in the workers’ memories regarding their working hours. Additionally, we were able to examine not only the relationship between the monthly/total working hours and workers’ health, but also the associations between the frequency of exposure to long working hours and workers’ health. Nevertheless, the study participants were recruited from one company. This limits the generalizability of our findings. Our cohort project is ongoing and we are collecting data from several other companies. We look forward to examining these other sources of data. Although we used the company’s automatic records to determine the time at which the workers entered and left the office, the participants were asked to report their start and end times of work to the company when they went home directly from customer visits. The reported timings of work were reviewed by the company and then registered in the attendance management system if those figures were judged as reasonable. Another essential limitation is that an approximate 6-month follow-up may have been insufficient for studying the prospective associations between long working hours and health endpoints. Obviously, continued follow-up must be performed for several years. In addition, the outcome measures were evaluated within a period of 1 year. Although we statistically adjusted for that time lapse, we did not know the specific changes that occurred in workers’ patterns during this period. Moreover, we were unable to adjust for the workers’ medication status as this information was missing. Instead, we adjusted for the previous results of health checkups. In addition, we were unable to collect sufficient data on smoking status, alcohol consumption, and exercise. Moreover, we defined the frequency of exposure to long working hours as the frequency of overtime work of 45 h or more per month. However, we were not able to account for the pattern of overtime work within a 6-month period. Sixty-four work patterns could possibly occur within the 6-month period. These patterns include the number of months with long overtime work and the number of months with no overtime work. We did not examine each of these patterns individually. Furthermore, the present study did not measure a couple of factors, such as working from home and commuting time, and it remained unclear how those factors would have affected the findings reported here.

## Conclusion

In conclusion, the associations between the respective monthly or average monthly work hours and health indicators were inconsistent. When assessing the associations between long working hours and workers’ health, working hours may thus need to be measured over a period of several months. The present study also showed that the averaged or frequent long working hours within the past 6 months may have a negative effect on LDL cholesterol levels.

## Data Availability

Research data are not shared.
